# Characterization of peanut phytochromes and their possible regulating roles in early peanut pod development

**DOI:** 10.1371/journal.pone.0198041

**Published:** 2018-05-25

**Authors:** Ye Zhang, Jinbo Sun, Han Xia, Chuanzhi Zhao, Lei Hou, Baoshan Wang, Aiqin Li, Min Chen, Shuzhen Zhao, Xingjun Wang

**Affiliations:** 1 Biotechnology Research Center, Shandong Academy of Agricultural Sciences; Shandong Provincial Key Laboratory of Crop Genetic Improvement, Ecology and Physiology, Jinan, PR China; 2 College of Life Science, Shandong Normal University, Jinan, PR China; East Carolina University, UNITED STATES

## Abstract

*Arachis hypogaea* L. geocarpy is a unique feature different from other legume plants. Flowering and fertilization occur above ground, while the following processes of pod formation and development proceed in the soil. The zygote divides only few times to develop into pre-embryo and then further embryo developmental process stops when the gynoecium is exposed to light condition or normal day/night period. In this study, eight phytochrome genes were identified in two wild peanuts (four in *Arachis duranensis* and four in *Arachis ipaensis*). Using RACE and homologous cloning, the full CDS of *AhphyA*, *AhphyA-like*, *AhphyB* and *AhphyE* were acquired in cultivated peanut. Protein structure analysis showed that the conservative coding domains of phytochromes from a number of other plant species were found in these proteins. The C-terminal of AhphyA, AhphyA-like and AhphyB could interact with phytochrome-interacting factor 3 in vitro. The expression patterns of these genes in various tissues were analyzed by qRT-PCR, and significant differences were observed. Interestingly, the expression levels of *AhphyA-like* changed significantly during gynophore growth and early pod development. Furthermore, protein accumulation patterns of AhphyA and AhphyB in gynophore were different during early pod development stages in that AhphyA and AhphyB proteins were not detected in S1 and S2 gynophores, while significant accumulation of AhphyA and AhphyB were detected in S3 gynophore. These results provided evidence that phytochromes mediated light signal transduction may play key roles in peanut geocarpy development.

## Introduction

Peanut (*Arachis hypogaea* L.) is one of the most important oil crops with high protein content in the seeds. A unique feature of pod development in peanut is that flowering and fertilization are accomplished above ground, while the occurrence of pod formation is under the ground[1]. After fertilization, embryo-containing ovary elongates and grows downward to the ground, forming a peg-like structure. The elongating ovary pushes the tip of the ovary penetrated into the soil where embryo and pod development are initiated. Failure of soil penetration of the ovary leads to seed abortion under normal day/night period or continuous light condition. Upon soil penetration of the ovary, there are changes in the environmental conditions including light, mechanical stimuli, moisture and nutrition [2]. Previous studies showed that light is the most important factor to inhibit peanut embryo and pod developmental processes. Red light and white light inhibit the growth of peanut ovules, while far-red light and dark condition promote ovules enlargement [3]. These results indicated that phytochromes might participate in peanut embryo development process. Further study using immunocytochemistry technology indicated that the protein levels of phytochrome change significantly before and after ovary soil penetration [4]. In this study, the author used antibody raised from Pea-25 which is able to detect a conserved epitope of three phytochromes (phyA, phyB and phyC)[5]. So far, there is no report on peanut phytochrome protein detection using peanut phytochrome antibodies.

Arabidopsis phytochromes are well-characterized photoreceptors, which detect far-red light and red light, and participate multiple processes of plant growth and development [6–8]. There are five phytochromes discovered in Arabidopsis, phyA-phyE, which regulate seed germination, de-etiolation, shade avoidance response, circadian entrainment and flowering [6, 9–11]. Phytochromes have two distinct conformations in vivo, the red light-absorbing inactive form (Pr) and the far-red light-absorbing active form (Pfr). Phytochromes are soluble proteins and covalently attached to a light-absorbing linear tetrapyrrole chromophore, phytochromobilin[12]. Through phytochromobilin perception of light signal, phytochromes convert between the two conformations. According to light stability, phytochromes were divided into two distinct groups, type1 and type2. Type1 is light labile (phytochrome A, phyA), more abundant in dark-grown seedlings. It degrades rapidly when transferred from dark to light condition [13, 14]. The degradation of phyA protein under light was regulated at transcription and post-transcription levels [14–16]. The type 2 phytochromes, phyB-phyE, are light stable, and almost unaffected by light. In Arabidopsis, phyB is the most abundant phytochromes in light grown seedling, while the content of phyC-phyE is low [13, 17]. Phytochromes display both synergy and antagonism roles in regulation of plant development [7, 18, 19]. phyA and phyB played important roles and co-regulated seed germination under far red (FR) and dark condition. Analysis of *phyAphyB* double mutant showed that phyB inhibit the germination of *phyA* mutant under FR condition [18]. In tomato, phyA and phyB play antagonistic function during cold tolerance. In this process, active phyA can induce the expression of CBF gene through accumulating ABA and JA content to response cold treatment, while phyB is involved in negative regulation of CBF-mediated cold tolerance through ABA and JA signaling [19].

Phytochromes were synthesized in the cytosol in the inactive form (Pr). Light-regulated nuclear localization of phytochromes was the key event in phytochrome signaling cascade. After being exposed to continuous red light, phyB is accumulated in nuclear through its nuclear localization signal (NLS) in the C-terminal [20]. Both the localization mechanism and the location of phyC, phyD and phyE subcellular localization were similar to phyB [21]. Nuclear localization of phyA is distinct from that of phyB. phyA does not contain a typical NLS indicating the existence of other transport machinery including far-red elongated hypocotyl 1 (FHY1) and FHY1-like (FHL) which play important roles in phyA nuclear localization [22–24]. In nuclear, phytochromes regulate the expression of target genes through the interaction of other interacting proteins. Phytochrome-interacting factors (PIFs), belonging to bHLH transcription factor family, are the negative regulators of phytochrome signaling, which promote skotomorphogenesis and repress photomorphogenesis [25, 26]. PIF transcription factors are capable of binding directly to promoters of target genes to active/repress gene expression. The protein abundance of PIFs is regulated by phytochromes under light treatments, through phosphorylation and ubiquitination, and degraded via 26S proteasome [27–30]. PIFs are also key mediators of light signal transduction and phytohormone signaling to modulate plant growth and development. DELLA is the negative regulator of GA signaling, and is able to physically interact with PIF3 and PIF4 [31]. The interaction between DELLA and PIFs leads to the repression of PIFs binding to promoter sequence of their target genes which promoting cell elongation[32]. PIF4 could may interact with BZR1 to regulate transcription of PRE-class of bHLH transcription factors, positive regulators of plant growth [33]. Hundreds of light- and BR-responsive genes are co-regulated by BZR1 and PIF4 through the same target sequence (CGTG), and this co-occupancy mechanism allows PIF4-BZR1 complex to modulate plant growth and development in response to light and BR signaling. Ethylene signal and light signal pathways are connected by EIN3 which can bind to PIF3 promoter to activate its transcription in light[34]. The crosstalk between light and phytohormones co-regulate plant growth and adaptation to different environmental conditions.

Light signal pathway has been relatively thoroughly studied in Arabidopsis, including components, function of each partners and signal transduction mechanism. Light signal plays a critical role for early peanut pod formation and development. However, the molecular mechanisms how light signal affecting embryo development has not been well characterized. In this study, we identified the phytochrome family genes in wild peanut, cloned the full-length cDNA sequences of phytochrome family members in cultivated peanut, predicted the protein structure and performed interaction assay between phytochromes and PIF3 using yeast-two-hybrid. We also analyzed gene expression and protein accumulation patterns during peanut early pod development.

## Materials and methods

### Data collection and identification of phytochrome family

Protein sequences of five members of *Arabidopsis thaliana* phytochrome family were obtained from the Arabidopsis Information Resource (TAIR) database (http://www.arabidopsis.org/). ID of these protein sequences were listed in S1 Table. The Hidden Markov Model (HMM) of the conserved key domains of five phytochromes was analyzed using the Pfam online software (Pfam 29.0) (http://pfam.xfam.org/).

The conserved key domains of N-terminal of phytochromes are the GAF domain and PHY domain with HMM ID as pfam01590 and pfam00360 in Pfam database. The conserved key domain of C-terminal of phytochromes is HisKA domain with HMM ID as pfam00512 in Pfam database. The amino acid sequences of three HMMs were used to identify all possible phytochrome amino acid sequences in the genomes of two wild species (*Arachis duranensis* and *Arachis ipaensis*) (http://www.peanutbase.org/) using blastp (E<0.001). The names of wild peanut phytochromes were determined by the best hit proteins. Protein sequences of other species were collected from UniProt (http://www.uniprot.org/), Genbank (http://www.ncbi.nlm.nih.gov/genbank/) and Phytozome (https://phytozome.jgi.doe.gov/pz/portal.html) and listed in S1 Table.

The chromosomal locations of phytochromes in wild peanuts were acquired from peanut genome database. The gene structures of phytochrome genes in wild peanut were analyzed using Gene Structure Display Server 2.0 online software (http://gsds.cbi.pku.edu.cn/)[35].

### Plant materials

A cultivated peanut Luhua-14 was used in this study. Peanuts were grown in the experimental farm of Shandong Academy of Agricultural Sciences. Seeds were sown in May and harvested in September. Plant materials were collected from plants of about 60 DAG (days after germination), including root, stem, leaf and flower. Three developmental stages of peanut pegs were used in this study [36]. Aerial grown pegs which were green or purple in color with the length of 3–5 cm was assigned as S1; pegs grown in soil for about 3 d were white in color and with no detectable ovary enlargement were assigned as S2; pegs buried in soil for about 9 d with very small enlarged ovary were assigned as S3. Tissues were frozen in liquid nitrogen immediately after cut from the plants for RNA and protein extraction. Seeds were germinated under dark condition on humid filter paper at 28°C for 5 d. Then the seedlings were transferred to white light for different periods. Peanut hypocotyl was collected and frozen in liquid nitrogen immediately for protein extraction. Overexpression constructs of *AhphyA* and *AhphyB* were transformed to Arabidopsis by floral dipping method. F2 homozygous plants were planted in greenhouse with the temperature of 20°C and 16h/8h (light/dark) light cycle for 40 d. The rosette leaves were collected and immediately frozen in liquid nitrogen for protein extraction.

#### Gene cloning and sequence analysis of phytochrome genes in cultivated peanut

CDS fragments of *AhphyA*, *AhphyB* and *AhPIF3* were extracted from transcriptome sequences in GenBank. The full-length of *AhphyA* (KP311317), *AhphyB* (KP311316) and *AhPIF3* (KT984758) were amplified using 3' RACE and 5' RACE. One homolog *AhphyA* sequence, named *AhphyA-like* (KT984757), was isolated by comparing *AhphyA* cDNA to peanut database (http://www.peanutbase.org). We obtained the full-length of *AhphyE* by homologous cloning strategy. The full-length sequences of *AhphyA-like* and *AhphyE* were isolated by RT-PCR-based method from peanut flower. The primer sequences used to amplify the full-length of *AhphyA*, *AhphyA-like*, *AhphyB*, *AhphyE* and *AhPIF3* were listed in S2 Table. PCR amplifications were carried out using pfu (TransGen Biothech, China) and the program was 94°C 5 min; 94°C 30 s, 56°C 30 s, 72°C 3.5 min for 32 cycles; and 7 min at 72°C. PCR products were purified by agarose gel recovery kit (TIANGEN Biotech, China). The full-length cDNA was ligated to pMD18-T vector (TaKaRa, China) and sequenced by Invitrogen Co.Ltd. (Beijing, China). The sequences were analyzed using DNAMAN software. We used Expasy online software to analysis the protein isoelectric point (pI) and molecular weight (Mw) of phytochromes in cultivated peanut (http://web.expasy.org/compute_pi/). The conserved domains of AhphyA, AhphyA-like AhphyB and AhphyE were predicted by Pfam online software (Pfam 29.0) (http://pfam.xfam.org/). The protein structure models of these phytochromes were obtained using Illustrator for Biological Sequences (IBS 1.0) online software (http://ibs.biocuckoo.org/).

### Multiple sequence alignments and phylogenetic analysis

Multiple sequence alignments were carried out by ClustalW2 online software (http://www.ebi.ac.uk/Tools/msa/clustalw2/) using the full-length amino acid sequences of phytochromes in peanut and other species. The phylogenetic trees were constructed using Neighbor-Joining (NJ) method by MEGA 7.0 software. To analysis the classification of subgroups of peanut phytochromes, seven *G*. *max* phytochromes, two *L*. *japonicus* phytochromes, three *M*. *truncatula* phytochromes, two *P*. *sativum* phytochromes, five *A*. *thaliana* phytochromes, six *B*. *rapa* phytochromes, five *S*. *lycopersicum* phytochromes, eleven *S*. *tuberosum* phytochromes, two *N*. *tabacum* phytochromes, six *Z*. *mays* phytochromes, three *S*. *bicolor* phytochromes and three *O*. *sativa* phytochromes were used for phylogenetic analysis. The bootstrap analysis generated 1,000 replicates to calculate the relationship. All phytochrome IDs were listed in S1 Table.

### Vector construction and yeast two-hybrid analysis

The cDNA sequences encoding the C-terminal of AhphyA (601–1125 aa), AhphyA-like (601–1125 aa) and AhphyB (626–1151 aa) were cloned into pGADT7 at NdeⅠ/SmaⅠ, NdeⅠ/SmaⅠ, BamHⅠ/XhoⅠ restriction sites, respectively. Full length of AhPIF3 with the removed N terminal 101–150 aa was cloned into pGBKT7 at EcoRⅠ/ BamHⅠ restriction sites. The primers used for these sequence amplifications were listed in S2 Table. The matchmaker two-hybrid system (Clontech, CA) was used for protein interaction analysis.

### RNA extraction and gene expression analysis

Total RNA was extracted from root, stem, leaf, flower, S1, S2 and S3 gynophores using Trizol Reagent (TaKaRa, Dalian, China). mRNA was isolated and purified using DNase I to degrade DNA contamination. The 1st strand cDNA was synthesized using Primer-ScriptTM 1st Strand cDNA Synthesis Kit (TaKaRa) and oligo (dT) primer. Gene-specific primers for qRT-PCR analysis of *AhphyA*, *AhphyA-like* and *AhphyB* were designed by Primer 5.0 software. The primer sequences were listed in S2 Table. FastStart Universal SYBR Green Master Mix (Roche, USA) was used for qRT-PCR. The reaction mixture was consisted of 2 μL of 50 x diluted 1st cDNA, 0.5 μL of 10 μmol of each primer and 10 μL 2×FastStart Universal SYBR Green Master Mix. An ABI 7500 real-time PCR system was used under the following program: 95°C for 10 min, then 40 cycles of 95°C for 15 s and 60°C for 1 min. Peanut actin was used as the reference gene for normalization. Non-specific products were identified by melting curve analysis. The ^2-△△^CT method was used to analyze the relative expression level [37].

### Total protein extraction and western-blot analysis

Samples (0.5 g) were ground in liquid nitrogen and immediately transferred to Eppendorf tubes in the presence of 1 ml protein extraction buffer (100 mM Tris-HCl (pH 7.5), 5 mM EDTA (pH 8.0), 0.2% Mercaptoethanol, and 100 mM PMSF). After 30 min on the ice, the mixed liquid was centrifuged at 12,000 rpm for 10 min at 4°C. The supernatant was transferred to a new Eppendorf tube and centrifuged at 12,000 rpm for 20 min at 4°C. The supernatant was transferred to a new Eppendorf tube and SAS (the supernatant of 100 ml Milli-Q containing 75 g ammonium sulfate overnight at 4°C) was added and mixed (supernatant: SAS = 2:3). After 30 min on ice, the mixture was centrifuged at 12,000 rpm for 30 min. Precipitate was dissolved in 10% of the original volume of protein extraction buffer. Protein concentration was measured by Bradford Method, and the value of OD_595_ was recorded. Proteins were separated on 10% SDS-PAGE and transferred onto PVDF membrane (Millipore). The membrane was blocked overnight with 1% BSA blocking buffer, and then probed with the polyclone rabbit antibody against phyA, phyB and actin. Anti-phyA and Anti-actin polyclonal antibodies were purchased from Agrisera (Sweden) and Sangon biotech (China), respectively. Peptide (ATNRVPHHQHQNQHC) were synthesized (GenScript, China) corresponding to the N-terminal region to peanut phytochrome B and used for Anti-phyB polyclonal antibody development. 1000 × diluted antibodies were used in this study. After washing for three times by TBST buffer, the membrane was incubated in the secondary antibody, goat anti-rabbit antibody (Sangon biotech, China) (1000 × dilution) at room temperature for 2 h. Hybrid signals were visualized by DAB color kit (Sangon biotech, China).

## Results

### Identification of phytochrome genes in wild peanuts

**Fig 1:**
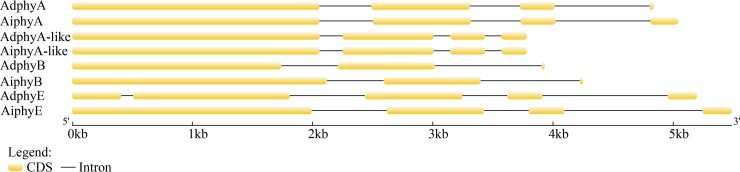
Gene structure of phytochrome genes in wild peanuts.

Eight phytochrome genes were identified in wild peanuts, four from *A*. *duranensis* and four from *A*. *ipaensis*. The detailed information of these phytochrome genes was listed in Table 1. Two *phyA*, one *phyB* and one *phyE* were discovered in each species. *phyA*, *phyA-like* and *phyB* were distributed on the same chromosome from both *A*. *duranensis* and *A*. *ipaensis*, while *phyEs* were distributed on different chromosomes in the two different species. In wild peanuts, the lengths of these genes varied from 2574 bp to 3390 bp and the lengths of proteins varied from 858 aa to 1130 aa. The numbers of exon were the same in phyA, phyA-like and phyB between *A*. *duranensis* and *A*. *ipaensis*, while the numbers of phyE exon were different in these two species (Fig 1).

**Table 1:** Members of phytochrome identified in wild peanuts.

Gene Name	Gene ID	Species	Chromosome Location	ORF (bp)	Amino Acid	Exon Numbers
*phyA*	Aradu.G6IAK	*A*. *duranensis*	A09:20644861..20651961	3195	1065	4
*phyA*	Araip.K62H2	*A*. *ipaensis*	B09:26116014..26122370	3390	1130	4
*phyA*- like	Aradu.E3ZED	*A*. *duranensis*	A06:6070225..6074408	3315	1105	4
*phyA*- like	Araip.HY5UP	*A*. *ipaensis*	B06:10971497..10975711	3315	1105	4
*phyB*	Aradu.T66QJ	*A*. *duranensis*	A04:12842087..12847987	2574	858	3
*phyB*	Araip.BH6DK	*A*. *ipaensis*	B04:14188155..14194207	2952	984	3
*phyE*	Aradu.H9LWJ	*A*. *duranensis*	A06:35179509..35184800	3060	1020	5
*phyE*	Araip.70MBH	*A*. *ipaensis*	B03:65314031..65319952	3342	1114	4

### Cloning of phytochrome genes in cultivated peanut

The full-length of *phyA* and *phyB* in *A*. *hypogaea* were acquired using 3’ RACE and 5’ RACE. Based on homologous cloning, the full-length sequences of *AhphyA-like* and *AhphyE* were obtained. The CDSs of both *AhphyA* and *AhphyA-like* were 3378 bp and encoding 1125 aa; the complete CDS of *AhphyB* gene was 3456 bp and encoding 1151 aa; the full-length CDS of *AhphyE* gene was 3342 bp and the length of protein sequence was 1113 aa. The molecular weight of phytochromes in cultivated peanut ranged from 123.5 kDa to 128.2 kDa. The pI values of AhphyA and AhphyA-like were 6.01, while AhphyB and AhphyE have pI values of 5.76 and 5.72. The detailed information of phytochrome genes was shown in S3 Table.

### Sequence analysis of phytochromes in wild and cultivated peanuts

**Fig 2:**
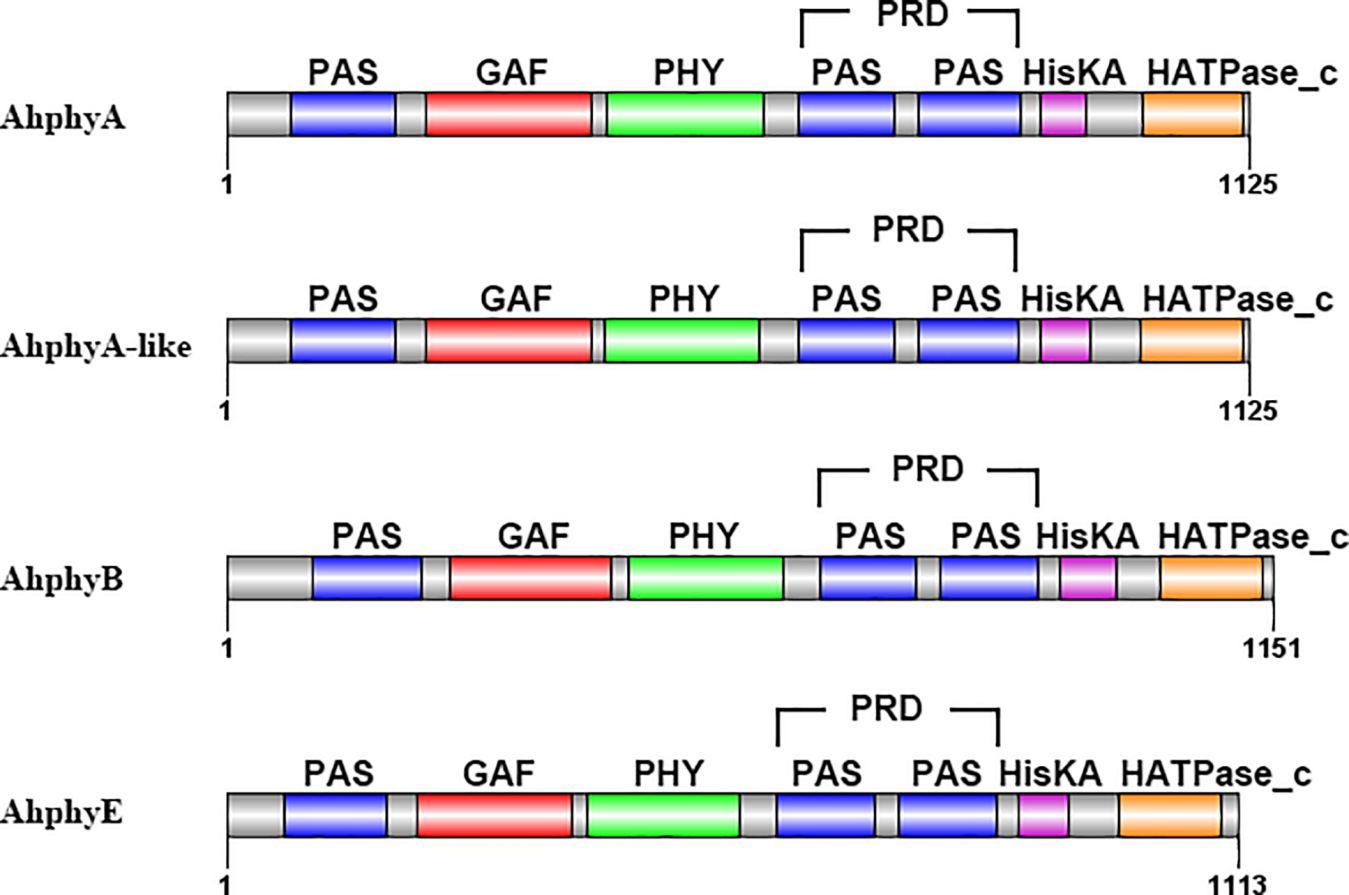
Predictions of conserved domains of AhphyA, AhphyA-like, AhphyB and AhphyE.

The structures of phytochromes were divided into two parts, N-terminal domain perceiving light signal and C-terminal transmitting light signal which connected by a flexible hinge region. Two phyA, one phyB and one phyE were identified in wild peanuts and cultivated peanut, respectively. The conserved domains and positions in their protein sequences were showed in Fig 2 and S4 Table. Three main domains (PAS, GAF and PHY) were present in the N-terminal of phytochromes and all three domains were found in phytochromes of wild and cultivated peanuts. Two domains in the C-terminal were the two PAS-repeats domain (PRD) and the histidine-related domain (containing HisKA and HATPase_c). There was no HisKA and HATPase_c domain in C-terminal of AdphyA. In wild peanuts, there was no HATPase_c domain in C-terminal of AdphyB and AiphyB. The PRD and histidine-related domains were all found in other phytochromes of wild and cultivated peanuts.

The protein sequences of phyA, phyB and phyE encoding by *A*. *hypogaea* showed higher similarity to sequences from *A*. *ipaensis* comparing to *A*. *duranensis*, while AhphyA-like showed higher sequence similarity to AdphyA-like than AiphyA-like (S5 Table). Phytochrome genes in *A*. *hypogaea* displayed higher similarity to genes from *A*. *ipaensis*.

**Fig 3:**
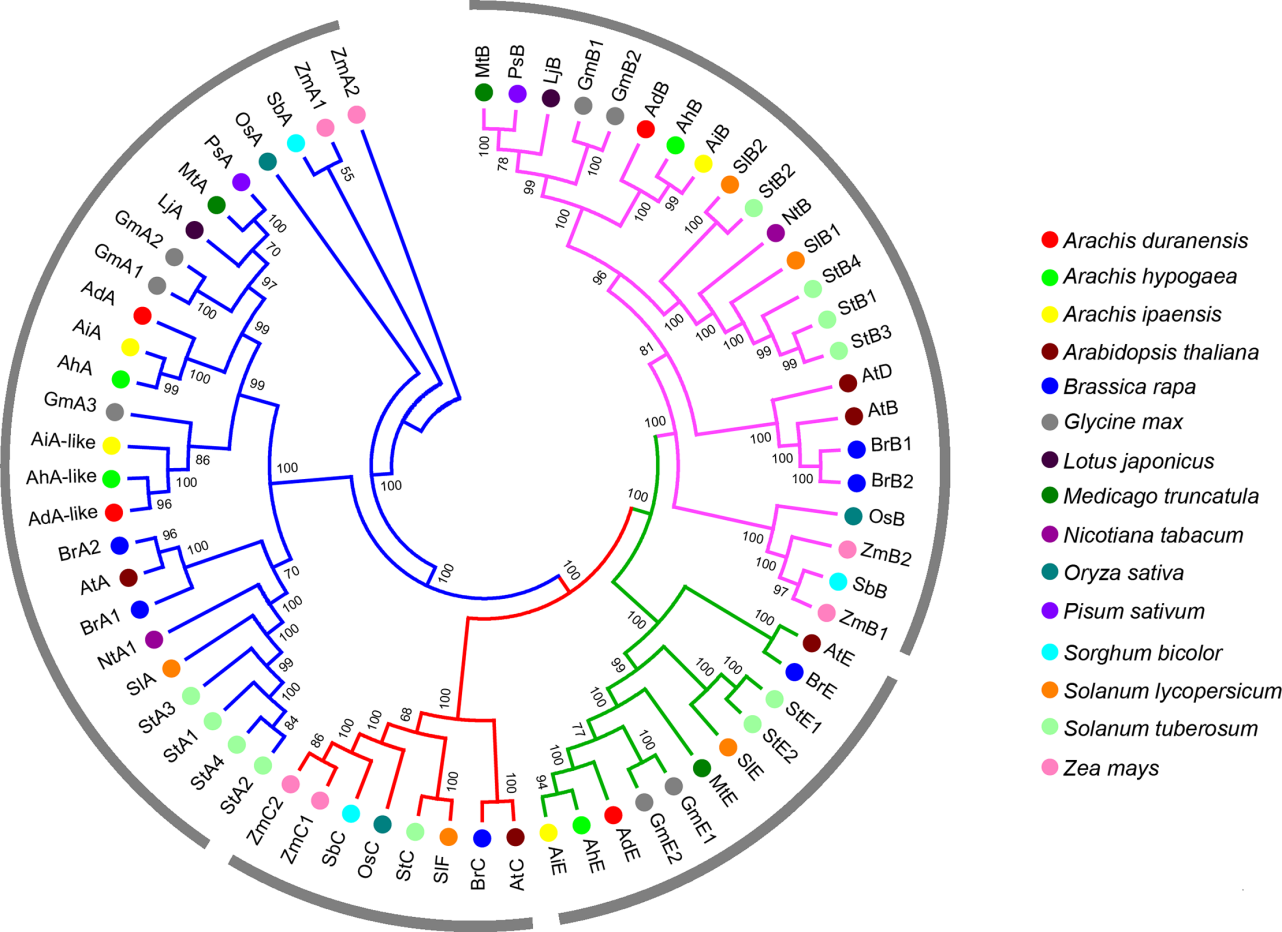
Phylogenetic analysis of plant phytochrome genes.

Full-length protein sequences of these 12 phytochrome genes from wild and cultivated peanuts and other species (*Arabidopsis thaliana*, *Brassica rapa*, *Glycine max*, *Lotus japonicas*, *Medicago truncatula*, *Pisum sativum*, *Solanum lycopersicum*, *Sola-num tuberosum*, *Nicotiana tabacum*, *Oryza sativa*, *Sorghum bicolor* and *Zea mays*) were used for phylogenetic tree-building (Fig 3). Phytochromes were divided into four sub-groups, including phyA, phyB, phyC and phyE sub-groups. In each sub-group, the phylogenetic relationships of wild peanut and cultivated peanut phytochrome genes were closer to legume species (*G*. *max*, *L*. *japonicas*, *M*. *truncatula* and *P*. *sativum*) than other dicotyledon and monocotyledon species. The sequences of phyA encoded by wild and cultivated peanuts were closely related to legume species phyA (GmphyA, LjphyA, MtphyA and PsphyA). The phyA-like of wild and cultivated peanuts appeared to be closer to GmphyA3, which was different from GmphyA1, GmphyA2 and phyAs from other three legume species. phyC sub-group were absent in wild and cultivated peanuts, and other legume species (*G*. *max*, *L*. *japonicas*, *M*. *truncatula* and *P*. *sativum*), suggesting that phytochrome C sub-group might be lost before speciation of these legumes.

### Expression of four phytochrome genes in different tissues

**Fig 4:**
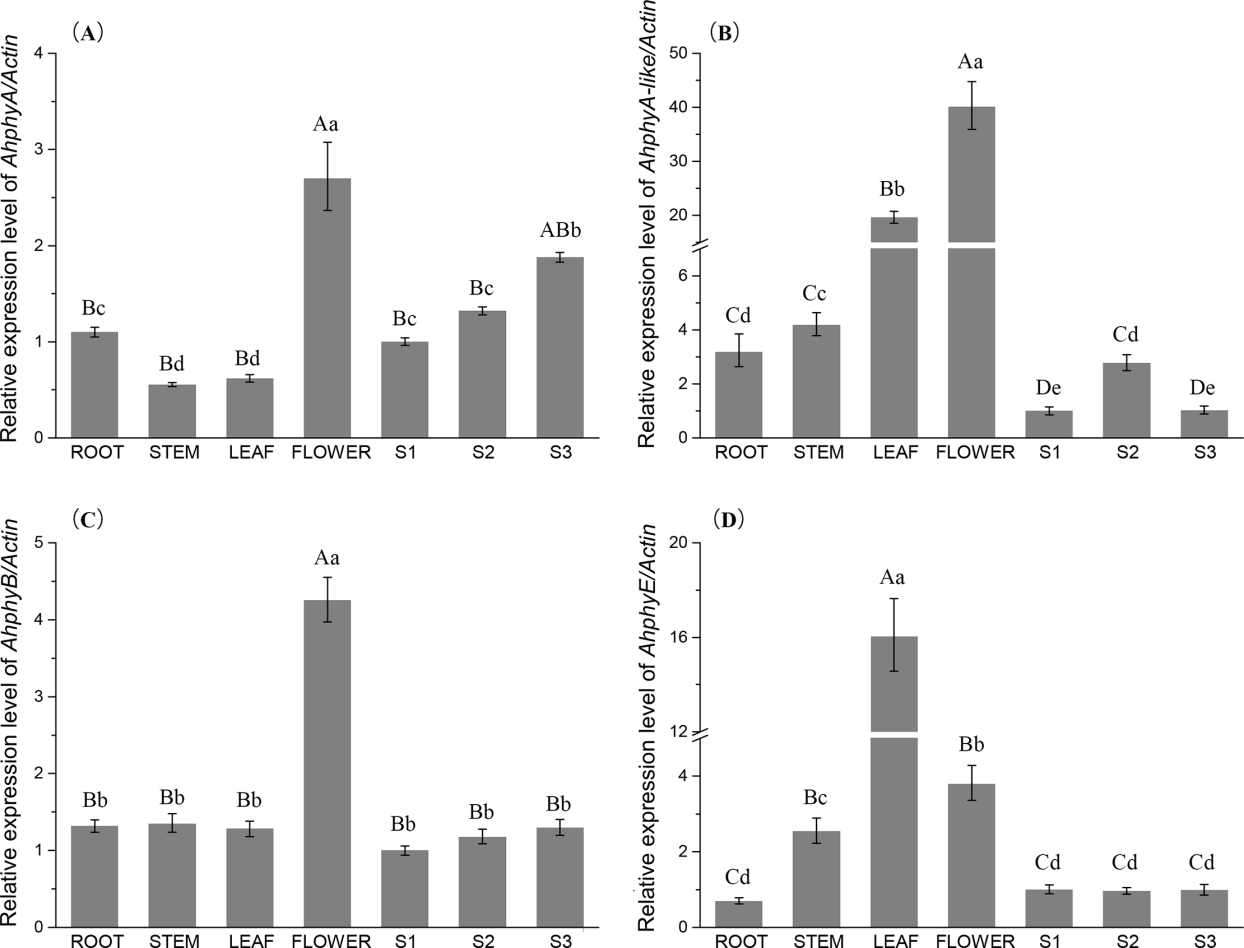
The relative expression levels of *AhphyA* **(A)**, *AhphyA-like* **(B)** *AhphyB* **(C)** and *AhphyE* **(D)** in different tissues and gynophores. It was shown as the expression fold change compared with S1 gynophore. Different lowercase letters indicate significance between differences in relative expression levels (P<0.05), different capital letters indicate very significance between differences in relative expression levels (P<0.01).

RT-PCR analysis was used to study the expression levels of peanut phytochrome genes in different tissues in order to understand their possible roles in plant development. Root, stem, leaf, flower, aerial gynophore (S1), white gynophore (S2) and swelling pod (S3) were collected from 60 d old field grown plants for expression study. The results indicated that *AhphyA*, *AhphyA-like*, *AhphyB* and *AhphyE* were expressed in all these tissues (Fig 4). The expression level of *AhphyA* was the highest in flower and the lowest in stem. The expression level of *AhphyA* in leaf was slightly higher than that in stem. In root, the expression levels of *AhphyA* were two-fold higher than that in stem. During peanut gynophore development, the expression level of *AhphyA* increased and reached the maximum in S3. *AhphyA-like* expressed at high level in leaf and flower, which was 20-fold and 40-fold higher than that in S1. The expression levels of *AhphyA-like* in root and stem were higher than that in S1. In S1 gynophore, the expression level of *AhphyA-like* was the lowest. The expression levels of *AhphyA-like* increased in S2 and decreased in S3. *AhphyB* gene expressed at the highest level in flower. The expression levels of *AhphyB* in root, stem and leaf were similar. The expression levels of *AhphyB* were similar in S1, S2 and S3. The expression level of *AhphyE* was the highest in leaves and the lowest in root. From S1 to S3, no significant difference was observed for *AhphyE* expression.

### Accumulation of phytochrome proteins in peanut pod

**Fig 5:**
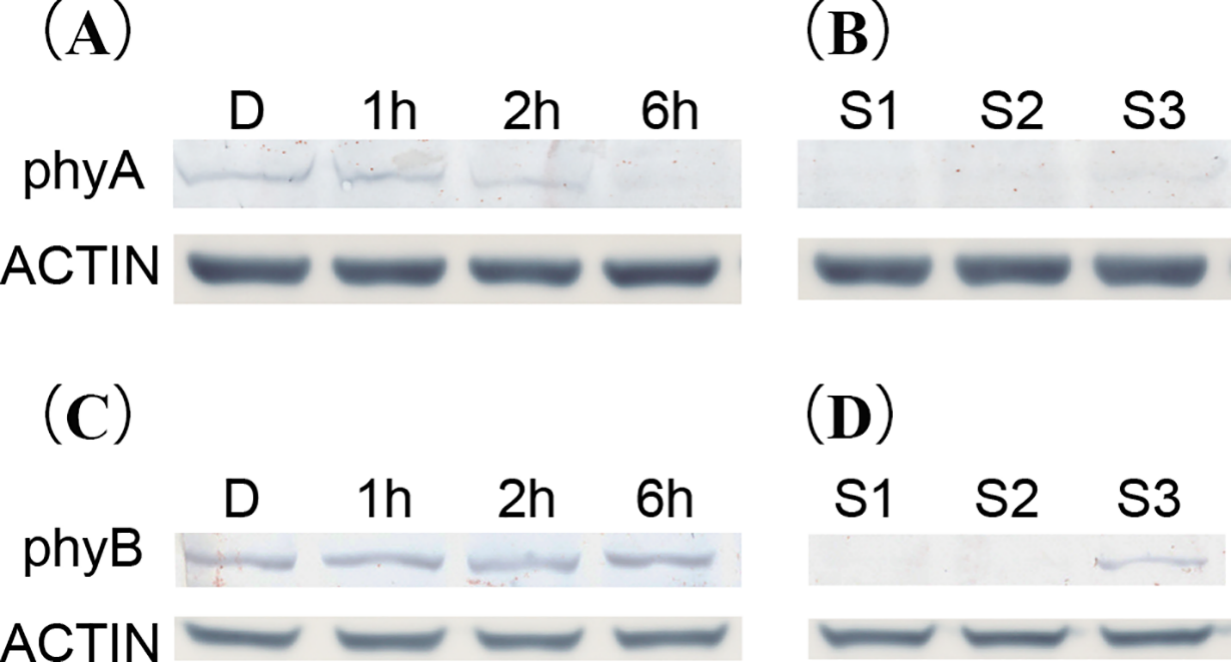
Western-blot analysis of phyA and phyB in peanut hypocotyl and different developmental stages of gynophores. **(A)** and **(C)** Accumulations of phyA and phyB in peanut hypocotyl under different light treatments; **(B)** and **(D)** accumulations of phyA and phyB in S1, S2 and S3 samples.

To verify the recognition capability of AtphyA and AhphyB antibodies, we tested antibodies firstly in Arabidopsis seedlings overexpressing AhphyA and AhphyB, respectively. Both antibodies could specifically recognized AhphyA and AhphyB in transgenic plants (S1 Fig). Using Arabidopsis phyA antibody, the accumulation of AhphyA in peanut hypocotyl was examined (Fig 5A and 5B). Results showed that peanut phyA was presented in dark grown seedlings and the level decreased to undetectable level after 6 h of light exposure. Using this antibody, we examined the AhphyA level in S1, S2 and S3 samples. AhphyA protein was not detected in S1 and S2, while very low level of AhphyA was detected in S3. Specific amino acid sequence of peanut AhphyB was synthesized and used for the antibody preparing. Under dark condition, AhphyB protein was accumulated in peanut hypocotyl (Fig 5C and 5D). AhphyB protein levels between dark- and light-grown hypocotyls were similar. After 6 h exposure to white light, AhphyB protein of peanut hypocotyl remained in a high level. Interestingly, AhphyB protein was not detected in S1 and S2, while in S3 when pods started to enlarge, significant level of AhphyB protein was detected.

### Interaction analysis between phytochromes and PIF3

**Fig 6:**
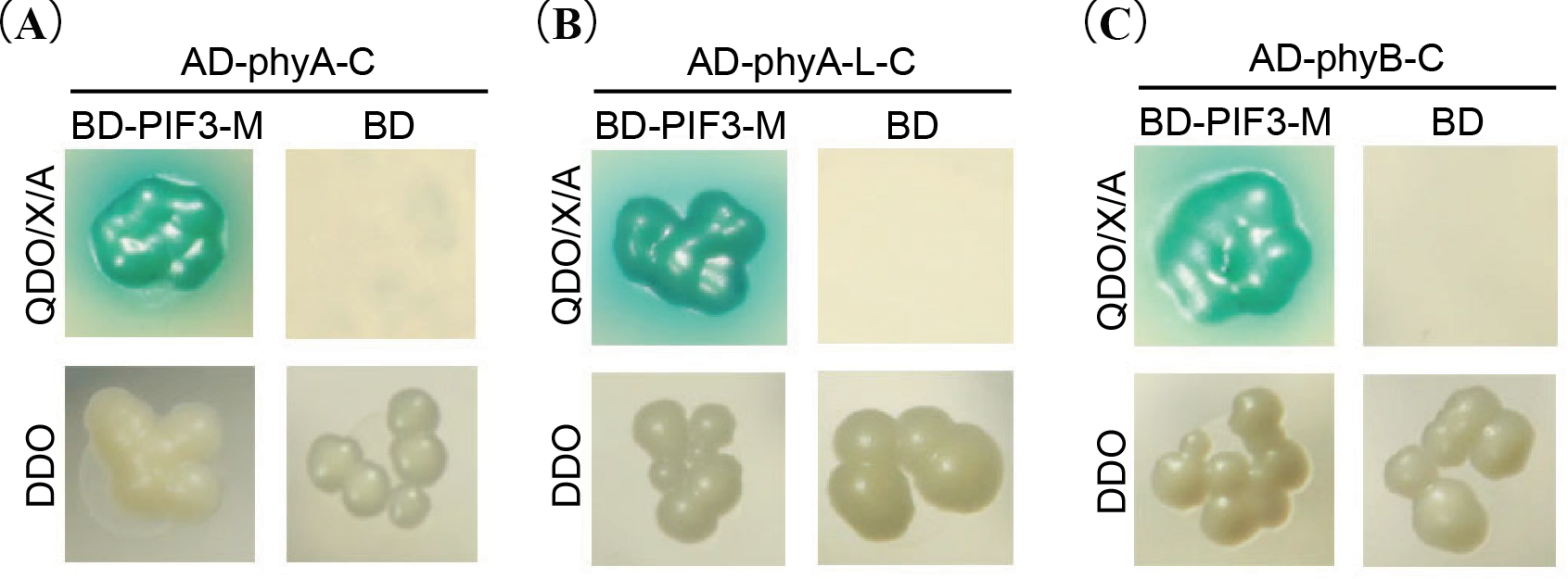
The interaction between the C-terminal of phytochromes and PIF3. **(A)** Interaction between phyA and PIF3; **(B)** Interaction between phyA-like and PIF3; **(C)** Interaction between phyB and PIF3.

To avoid self-activation, the C-terminal of phyA, phyA-like and phyB (named phyA-C, phyA-like-C and phyB-C) were cloned into pGADT7 vector. Previous study indicated that the self-activation region of phytochrome-interacting factors (PIFs) was at the N-terminal of 101–150 amino acids. Sequence of PIF3 without this region of the N-terminal (named PIF3-M) was cloned into pGBKT7. The interaction between phytochromes and PIFs was examined using yeast two-hybrid system. Yeast co-transformed with AD-phytochromes-C and BD-PIF3-M plasmids could grow normally on QDO/X/A through activating four reporter genes (Fig 6). However, the co-transformed yeast with AD-phytochromes-C and BD plasmids could not grow on QDO/X/A. These results indicated that phytochromes (phyA, phyA-like and phyB) could physically interact with PIF3.

## Discussion

After the formation of peanut gynophore in aerial, it grows downward. With the elongation, the tip region of the gynophore penetrates into soil and enlarges underground. Failure of soil penetration, peanut gynophore can continuously elongate, but pod development is repressed. Earlier study indicated that phytochromes sensed light signal to regulate peanut embryo growth and development in vitro [3]. Immunoblotting analysis suggested that phytochrome was localized in embryo and adjacent integument tissues, after gynophore was buried in soil for 8–12 days [4]. In this study, we cloned the full-length CDS of *AhphyA*, *AhphyA-like*, *AhphyB* and *AhphyE*. Conserved protein domain analysis showed that PAS DOMAIN, GAF DOMAIN and PHY DO-MAIN were presented in the N-terminal of AhphyA, AhphyA-like, AhphyB and AhphyE, while several motifs were different among them.

Phytochromes could be divided into two parts, N-terminus for specific photo-sensory and C-terminus for signal transduction. After photoconversion of the phytochrome to its active form (Pfr), phytochrome is capable to regulate gene expression. PIF3, a putative transcription factor and phytochrome signaling component, is interacted with the non-photoactive C-terminal domains of phyA and phyB [30, 38]. Mutations in the C-terminal domains of phyA and phyB disrupt the signal transduction from the photoreceptors to components of the transduction pathway, without affecting light signal perception [39, 40]. Using yeast-two-hybrid system, the physical interaction between the C-terminal of phytochromes (including AhphyA, AhphyA-like and AhphyB) and PIF3 were confirmed. Nevertheless, the interaction between the N-terminal (amino acids 1–600 of phyA and phyA-like, 1–625 of phyB) of these phytochromes (added PCB) and PIF3 were not detected (data not shown). In Arabidopsis, PIF3 bound more strongly to full-length Pfr form of phyB than that to N -terminal fragments (amino acids 1–644) in its Pfr form and non-photoactive form of C-terminal fragments (residues 645–1,211) of phyB[30, 38, 41]. These results indicated that each part of the protein is synergistic in the full-length molecule. No detectable interaction between N-terminal of phytochromes and PIF3 might be due to the truncated sequences.

In Arabidopsis, the function of each phytochrome is clearly characterized throughout the whole life cycle [7, 26]. However, little is known about the function of phytochromes during gynophore and pod development of peanut. In the current study, we analyzed *AhphyA*, *AhphyA-like*, *AhphyB* and *AhphyE* gene expression levels in different tissues and in different developmental stages of gynophores. Expression of *AhphyA*, *AhphyA-like*, *AhphyB* and *AhphyE* under natural day/night condition was detected in peanut root, stem, leaf and flower. In Arabidopsis, the expression of phyA is regulated by light [16]. In peanut, after gynophore penetrated into soil, both *AhphyA* and *AhphyA-like* expression increased in their mRNA levels. With the prolonged stay of gynophore in dark soil, *AhphyA* expression was up-regulated while *AhphyA-like* expression was down-regulated. The different behavior of these two genes at transcription level in S3 suggested their functions might be different in the initiation of peanut pod and embryo development. However, there was no change of *AhphyB* and *AhphyE* expression from S1 to S3. This result indicated that the expression level of phyE was consistent with that of phyB which was not regulated by light [16].

The unique characteristic of peanut is geocarpy development and light signal is an important factor that changed significantly during this process [42]. Previous studies indicated that phytochromes play crucial roles in pod formation [3, 4]; however, the specific regulatory mechanism is still unknown. The half-life of phyA in Arabidopsis hypocotyl was about 1–2 h at light condition. AtphyA protein can accumulate at high level in etiolated seedlings and decrease significantly when exposed to light [14], while AtphyB protein is relatively stable in de-etiolated seedlings [13]. When exposed to white light, AhphyA and AhphyB proteins in peanut hypocotyl behaved in the same trends as in Arabidopsis. Protein accumulation patterns of AhphyA and AhphyB in S1, S2 and S3 samples were significantly different. No accumulation of AhphyA and AhphyB proteins was detected in S1 and S2. Low level of phyA protein accumulated in S3, while abundant AhphyB protein was detected in S3. These results were consistent with previous study in which the presence of phytochrome is detected in ovular after gynophore soil penetration for 8–12 d [4]. From the protein accumulation results of AhphyA and AhphyB in peanut hypocotyls and gynophores, it was speculated that AhphyA and AhphyB protein accumulation in S3 gynophores was not resulted from light regulation. These results indicated that both AhphyA and AhphyB may participate in the regulation of peanut pod swelling. Although S3 gynophore was under dark condition, mostly AhphyB act as non-phosphorylation status. This was consistent with that cytoplasmic phyB participate cytoplasmic events of phytochrome signaling [7]. Therefore, both AhphyA and AhphyB could make a great contribution to peanut gynophore enlargement under dark condition.

## Supporting information

S1 Fig The accumulations of AhphyA and AhphyB in transgenic Arabidopsis lines. (TIF)

S1 Table Phytochrome genes identified in variety of plant species. (XLSX)

S2 Table Gene-specific primers used in this study. (XLSX)

S3 Table Members of phytochrome in *A*. *hypogaea*. (XLSX)

S4 Table Domains of phytochromes in wild and cultivated peanuts. (XLSX)

S5 Table Gene identities of wild and cultivated peanut phytochrome orthologous pairs. (XLSX)
