# Supplementary figures and images for: Characterization of peanut phytochromes and their possible regulating roles in early peanut pod development
Source: PLoS One. 2018 May 25;13(5):e0198041. doi: 10.1371/journal.pone.0198041 (PMC5969742; doi:10.1371/journal.pone.0198041)

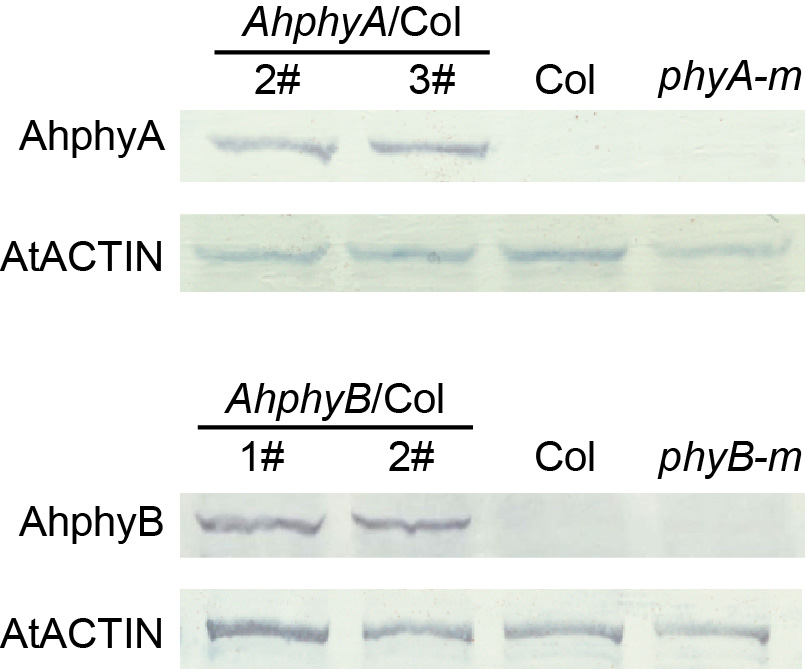

Supplement: S1 Fig — (TIF) [file pone.0198041.s001.tif]
